# Perfectionistic Self-Presentation and Suicide in a Young Woman with Major Depression and Psychotic Features

**DOI:** 10.1155/2014/901981

**Published:** 2014-09-24

**Authors:** Sabrina Hassan, Gordon L. Flett, Rohan Ganguli, Paul L. Hewitt

**Affiliations:** ^1^Centre for Addiction and Mental Health, York University, 1001 Queen Street West, Unit 4-1, Toronto, ON, Canada M6J 1H4; ^2^York University, 4700 Keele Street, Technology Enhanced Learning Building, 5022K, Toronto, ON, Canada M3J 1P3; ^3^Centre for Addiction and Mental Health, University of Toronto, 1001 Queen Street West, Unit 4-1, Toronto, ON, Canada M6J 1H4; ^4^University of British Columbia, 2136 West Mall, Vancouver, BC, Canada V6T 1Z4

## Abstract

A woman in her midtwenties with a history of major depressive disorder and a recent major depressive episode with mood-congruent psychotic features died by suicide. Two weeks before her death, she demonstrated exceptional elevations on the nondisplay of imperfection factor of Hewitt and Flett's Perfectionistic Self-Presentation Scale. Perfectionism and especially perfectionistic self-presentation have been strongly associated with suicide across several populations, accounting for unique variance in suicidality beyond depression and hopelessness. Yet interpersonal facets of perfectionism are not recognized as clinical risk factors for suicide. There is also a paucity of research on perfectionism in relation to psychotic symptoms. This case account illustrates the role of perfectionistic self-presentation in suicides that occur seemingly without warning and, to our knowledge, this is the first examination of perfectionistic self-presentation and suicide in a case where psychotic features occurred. This study, though single case-based, draws attention to perfectionism and perfectionistic self-presentation and their potential roles in suicide, especially when accompanied by other risk factors. Future research in this area may elucidate the role of perfectionism in suicide, singularly and in the context of a comprehensive clinical risk assessment, demonstrating whether perfectionism confers information about suicide risk beyond known clinical risk factors.

## 1. Introduction

Perfectionism has been characterized as “destructive” [1, 2] based on its associations with psychopathology and maladaptive outcomes, including personality disorders, anxiety, drug and alcohol abuse [3], depression [4], shame [5, 6], and suicide [1, 2, 7]. Research has demonstrated that the social aspects of perfectionism, specifically socially prescribed perfectionism and perfectionistic self-presentation, are associated most strongly with maladaptive outcomes (e.g., [8]). Although trait perfectionism and perfectionistic self-presentation have been strongly associated with suicide across a number of populations, the interpersonal facets of perfectionism are not yet clinically accepted risk factors for suicide, nor have they been examined in relation to psychotic symptoms or experiences.

Socially prescribed perfectionism is the perception that significant others have “unrealistic standards for one, evaluate one stringently, and exert pressure on one to be perfect” [3, p. 457]. Perfectionistic self-presentation accounts for unique variance (beyond trait perfectionism) and is the interpersonal expression of perfectionism, conceptualized as wanting to appear flawless in order to avoid negative evaluations from others [9]. Those who are high in perfectionistic self-presentation are less likely to reveal mistakes because of the inherent implication of their own defectiveness, the potential for negative evaluation from others, and subsequent loss of social approval [9]. Empirical evidence supports perfectionistic self-presentation as being comprised of three factors: perfectionistic self-promotion, or asserting and displaying one's perfection; nondisplay of imperfection, or hiding behavioral manifestations of one's imperfection; and nondisclosure of imperfection, or avoiding verbalizations of one's imperfection [9].

Psychotic symptoms are established risk factors for suicide, with 20–40% of affected individuals attempting suicide in their lifetime and 10–15% dying by suicide [10]. The risk for attempted suicide is the highest in the year following the onset of psychotic symptoms and remains elevated for the subsequent 7–10 years [11]. Trait perfectionism and perfectionistic self-presentation have been strongly associated with worsened psychopathology and maladaptive outcomes across a number of populations, but there is a relative paucity of research on perfectionism and psychotic symptoms (for exceptions, see [12, 13]). Hassan et al. [14, 15] recently found that perfectionism and especially perfectionistic self-presentation were related to worsened symptomatology and poorer functioning in those who experienced psychotic symptoms (specifically, those with schizophrenia, schizoaffective disorder, bipolar I disorder, major depressive disorder with psychotic features, and psychosis not-otherwise-specified). Additionally, Fialko and colleagues [16] reported that low self-esteem and negative evaluation beliefs about self and others were related to suicidal ideation in persons with psychotic illnesses. Thus, an argument can be made for potentially increased suicide risk in perfectionistic individuals who recently developed psychotic symptoms.

To our knowledge, this case study is the first examination of perfectionistic self-presentation and suicide. This study, though single case-based, seeks to stimulate discussion and draw clinical and research attention to trait perfectionism and perfectionistic self-presentation as potential clinical risk factors for suicide. We also seek to raise the question of whether perfectionism plays a unique role in those with psychotic symptoms. Informed consent for publication of case material was obtained from the next of kin, per institutional research ethics board recommendations.

## 2. Case Presentation

A woman in her midtwenties was hospitalized for the first time for suicidality in the context of a severe major depressive episode with mood-congruent psychotic features. She had a diagnosed major depressive disorder, but this was her first experience of psychotic symptoms. Several weeks prior to hospitalization, she had demonstrated a significant decline in functioning, with worsening depressed mood, disturbed sleep and appetite, severe anxiety, and wishes to end her life. She expressed low self-worth and significant delusional guilt over having committed a crime that would have dire consequences for those around her.

This individual's medical history was significant for migraine headaches, fibromyalgia, and Wolff-Parkinson-White syndrome. One year prior to admission, she lost her romantic partner to terminal illness. This loss was traumatic for her and soon after she developed a severe depression with emergent psychotic symptoms. The immediate family history was significant for suicide, with one parent dying by suicide and two siblings attempting suicide several times. Several first- and second-degree relatives were diagnosed with bipolar disorder (clinical records did not specify whether bipolar I or bipolar II disorder) and schizophrenia.

A psychiatric evaluation suggested this individual had a history of major depressive disorder (MDD) with severe, recurrent episodes. Her diagnostic profile at the time of her hospitalization included a severe major depressive episode with mood-congruent psychotic features; eating disorder not-otherwise-specified; generalized anxiety disorder (GAD); and panic attacks. Her history of MDD and GAD dated back to her teens, and she had an additional diagnosis of attention-deficit hyperactivity disorder. She reported no alcohol or substance use, and she had no history of self-harm or suicide attempts. At the time of her hospitalization admission, her Global Assessment of Functioning (GAF; [17]) score was 30, indicating that her level of functioning and/or symptomatology were seriously impaired by hallucinations or delusions, and there was serious impairment in her communication or judgement, and/or she demonstrated serious impairment across nearly all functional domains.

After a 5-week hospitalization and medication stabilization, this individual was discharged from inpatient care to outpatient psychiatric and case management services. At the time of this transition, her GAF score was 55 (indicating moderate symptom severity and/or moderate functional impairment) and she reported no suicidal ideation or psychotic experiences.

Two weeks later, she participated in a research study and completed a prespecified set of measures, which were administered to all study participants. While the study was initially designed for those with schizophrenia and schizoaffective disorder, eligibility criteria were later expanded to include other psychiatric diagnoses in which psychotic symptoms may appear. The Brief Psychiatric Rating Scale [18] is a clinician-rated measure of psychopathology severity. On this measure, her total score was 42, which reflects symptoms in the very-mild to mild range; symptoms specific to schizophrenia rated 13 (very-mild range) and general symptoms rated 29 (mild to moderate range). The Scale for the Assessment of Negative Symptoms [19] is a clinician-rated measure of affective flattening, anhedonia/asociality, avolition/apathy, alogia, and attention. On this measure, her summary score was 17, which reflects symptoms in the moderate-marked range; her composite score was 54 (mild to moderate range). On the Calgary Depression Scale for Schizophrenia [20], which is a clinician-rated measure of symptoms of major depressive disorder in persons with schizophrenia, a score of at least 5 indicates a person is at risk for major depressive disorder. She scored 15, endorsing severe hopelessness and mild suicidal ideation. The Rosenberg Self-Esteem Scale [21] is a self-report inventory of positive and negative feelings about the self, purported to comprise global self-worth. Her self-esteem was incredibly low, rated 2 out of a possible 30 points. The Clinical Global Impressions-Severity Scale (CGI-S; [22]) and the Global Assessment of Functioning (GAF) are clinician-rated measures of overall symptom severity and level of functioning. Her GAF was rated 45, which is indicative of serious symptomatology and/or serious impairment in functioning. She rated 6 on the CGI-S, which indicates she was considered severely ill.

On an abbreviated 15-item version of the Multidimensional Perfectionism Scale [3, 23], she rated 13/35 for other-oriented perfectionism (her expectation of perfection in others), 12/35 for self-oriented perfectionism (her expectation of perfection in herself), and 11/35 for socially prescribed perfectionism (the perception that others expect perfection from her). Compared to published norms, scores on all of these facets are considered relatively low, suggesting this young woman was not a perfectionist. On an abbreviated 12-item version of the Perfectionistic Self-Presentation Scale (PSPS; [9]), she rated 14/28 for nondisclosure of imperfection and 16/28 for perfectionistic self-promotion, both of which, in consideration of published norms, also suggest this woman was not a perfectionist. On the third PSPS facet, however, she scored an exceptional 24/28 for nondisplay of imperfection, which indicates she was most certainly someone who was highly concerned about her imperfect behaviors being observable to others. Examples of items she strongly endorsed on the nondisplay factor include “I judge myself based on the mistakes I make in front of others” and “Failing at something is awful if other people know about it.”

Review of this individual's medical records indicates her clinical team experienced her as guarded and evasive. For example, in response to inquiries about her well-being, she once stated there was more to her inner experience, but she was not able to share this with others. Other times she would provide brief, indirect answers but decline to elaborate when prompted. She also made statements that were not supported by other aspects of her experience (e.g., she reported good appetite and eating well; her weight continued to decrease). In addition, she reported feelings of guilt over things she should have done, such as visiting loved ones when they were ill, not doing better in school, and being a burden to her family (with whom she lived). She also reported fears she would be “caught out as a bad person,” and felt responsible for her loved one's terminal illness. Despite objective evidence of her competence (e.g., cognitive testing), she reported lack of skill and fear of failure as barriers to her recovery. Her clinicians noted that she seemed unable to accept that cognitive testing suggested no significant impairment, feeling certain that something was wrong with her. Although attempts were made to facilitate self-compassion and a broader understanding of her past behaviors, this individual was also unable to consider these perspectives. There were several comments about her worry of others' judgment of her. One psychiatrist commented that her depressive symptoms seemed to have “crossed into the psychotic spectrum” at the time of her admission.

Less than one month following her discharge from inpatient services, this young woman died by suicide. Prior to her hospitalization, she told her family she had researched ways to die but was not sure how to carry out her plan; she was also curious as to why others' attempts at suicide had failed. For her own suicide, she used means that were highly lethal and took precautions to ensure she would not be discovered. In the week prior to her death, she was observed to be functioning well, taking care of her ill mother, and completing chores around the house, and her demeanor was described as “happy.” On the night she died, she advised her family she was going to visit a friend and she was described as apparently “happy” as she left. She turned off her cell phone, checked into a hotel, and ingested an unknown number of pills, which were taken from a family member's medicine cabinet. She then used a mask and tank of helium to asphyxiate herself. It was later discovered that this young woman had again spent time researching ways to die. A note was discovered with her body.

## 3. Discussion

Perfectionistic self-presentation has been linked with suicide and worsened psychopathology [8]. Socially prescribed perfectionism accounts for unique variance in suicidality after controlling for depression and hopelessness, two key predictors of suicide [24]. Although suicide risk is high in those with psychotic symptoms [10], particularly following the onset of psychotic symptoms [11], trait perfectionism and perfectionistic self-presentation have not yet been investigated in relation to suicide and psychotic symptoms. This woman died by suicide following her first psychiatric admission and her first experience with psychotic symptoms. She had no history of self-harm or suicide attempts. Although she admitted her wish to die, she consistently denied having a plan for taking her life. Yet, within a month of her discharge from inpatient services, this woman died by suicide after researching highly lethal means.

A review of this individual's clinical history suggests behavior consistent with perfectionistic self-presentation. For example, in the case of her weight decrease alongside her report of good appetite and eating habits, she seemed aware of the “right” answers to relevant questions. Although she was initially willing to acknowledge her wishes to die, she was reticent to explore these feelings in depth, perhaps because of the social stigma associated with suicide, the risk for longer hospitalization or rehospitalization, or the risk that someone might interfere with her plan. Her concern about others' judgment and her report of fears of being exposed as a bad person are also consistent with perfectionistic self-presentation and with Fialko and colleagues' [16] findings on negative evaluation beliefs about others, suggesting she may have feared loss of love or approval if she did not measure up to others' expectations.

Aspects of her history are also suggestive of trait perfectionism. Her fear of failure and belief of personal incompetence are consistent with perfectionistic beliefs and her extremely poor self-esteem, and with Fialko and colleagues' [16] findings on negative evaluation beliefs about self. Her inability to explore other understandings of her guilt suggests a rigidity in her beliefs; though this may be explained as fixed delusional guilt, it could also be indicative of cognitive inflexibility characteristic of perfectionists. Perfectionists often feel responsible for things that are actually uncontrollable, for instance, the terminal illness of her loved one (though her perceived responsibility could also be attributed to her depression). Finally, this woman's inquiry, though guarded, into “successful” ways of taking one's life, suggests that she needed to be perfect in this respect, as well, not wanting to endure a “botched” suicide and its aftermath.

Aspects of this case also fit with the broader perfectionism and suicide literatures. Her feelings of worthlessness and fear of being “caught out as a bad person” may be suggestive of characterological shame as described by Andrews and colleagues [25]. Her unwillingness to expose herself vis-à-vis her low self-disclosure is also consistent with shame [26] and perfectionistic self-presentation. Shame is highly correlated with perfectionism [5, 6] and suicide [27]. Her feeling that she was no longer useful in her life or to her family is consistent with research linking completed suicide and perfectionism with perceived burdensomeness to kin [28, 29]. The idea of psychiatric symptomatology crossing into the psychotic (versus neurotic or borderline) spectrum fits with Kernberg's [30, 31] framework of personality organization, potentially helping to explain how this individual's fear of being negatively evaluated may have contributed to her suicide. Specifically, her long-standing worries about others' opinions may have intensified with her worsening depression to the point of significant impairment, with her hopelessness about her lack of competence and her feeling of being a burden contributing to her decision to end her life. Her inability or unwillingness to disclose her inner experiences is consistent with previous literature (e.g., [32, 33]) linking low self-disclosure with completed suicides that occur seemingly without warning. Interestingly, her score on the nondisclosure factor of the abbreviated PSPS was not especially elevated; nevertheless, her clinical notes document substantial nondisclosure and self-concealment. Finally, this case is consistent with research demonstrating that perfectionistic evaluative concerns predict higher diagnostic comorbidity [34] and the general notion that perfectionism is linked with significant health problems.

Indeed, several factors in this woman's history may have contributed to her suicide. She had a family history of completed suicide and suicide attempts, and a personal history of major depressive disorder with a recent severe major depressive episode with mood-congruent psychotic features. Statistically, she was particularly vulnerable given that her suicide occurred within a year of her first experience with psychotic symptoms; this was her first psychiatric admission, and she had recently been discharged from the hospital. She had also experienced significant loss and was feeling worthless and hopeless about her future; hopelessness is an established predictor of suicide, irrespective of diagnosis [35, 36]. Her self-esteem, another predictor of suicide risk ([37]; see also [16]), was remarkably low, and she felt herself to be a burden to her family. She also selected a method of suicide that was decidedly lethal and took steps to ensure others would not be able to find her before she died.

It is difficult to know which of the above-mentioned factors figured most prominently in this young woman's decision to die. A more likely explanation is that the presence of a number of risk factors contributed to her death in an additive way, each compounding her risk and increasing the likelihood that she would consider and follow through with suicide. In the context of multiple risk factors, the contribution of excessive perfectionism may have similarly compounded her risk. The cognitive rigidity that characterizes perfectionists may have made it difficult to penetrate her hopelessness, diminished self-esteem, delusional guilt, and shame, leading her to view herself as permanently flawed and without any hope of redemption, making a positive contribution in the world, or living a fulfilling life. Perfectionistic cognitive rigidity may have also made it particularly difficult to cope with the loss of her partner, challenging her worldviews and beliefs about the world, which may have been intolerable and contributed to the development of her psychotic symptoms as an ego-preserving strategy. This lack of cognitive flexibility may have also made it difficult for her to consider other options once she had begun to contemplate suicide.

As alluded to earlier, what is particularly noteworthy about this case is that this woman's score on the PSPS nondisplay of imperfection factor was exceptionally high, especially in relation to her scores on all other perfectionism subscales. It is possible that this spike in her perfectionism profile may be instructive in understanding her suicide. That is, her strong endorsement of nondisplay of imperfection items suggests she could not tolerate having her mistakes and the flawed aspects of herself publicly visible. One wonders if this is why she chose such lethal means of dying, so as to ensure she would not have to face the shame of a “botched” suicide. She may have been mindful of her family's previous experience with completed suicide and may not have wanted her body to be visible to them in the family home, instead choosing to go to a hotel. She may have felt that her partner's death was a permanent and inescapable symbol of her imperfection, as she felt she was to blame for his death. As with many individuals who have recently been discharged from hospital for the first time, she also may have experienced difficulties resuming her usual routine and managing daily hassles she may have prior to her illness; she may have taken these difficulties to be further indications of her imperfection and been unable to tolerate the resultant shame. In addition, her interpersonal characteristics, particularly her guardedness and reticence to self-disclose, likely played a role in this individual's completed suicide, particularly in terms of precluding opportunity for intervention.

This woman's case, to our knowledge, is the first to examine perfectionistic self-presentation and its contribution to a completed suicide. In conjunction with the existing literature linking trait perfectionism and perfectionistic self-presentation with suicidality, this case suggests that future investigations into the relationship among suicide, trait perfectionism, and perfectionistic self-presentation are warranted. While this woman had a history of major depressive disorder and only her most recent episode presented with mood-congruent psychotic features, it is unclear what role her psychotic symptoms played in her suicide. It is noteworthy that this woman died by suicide several weeks after her first experience with psychotic symptoms, even if mood-congruent in nature. It is possible that her psychotic symptoms may have played a contributory role in this case of suicide and that perfectionism may play a role in the experience of psychotic symptoms (e.g., schizophrenia, schizoaffective disorder, bipolar I disorder, psychosis not-otherwise-specified). One study has found that there is a relationship between depressive schemas and psychotic content [38], which suggests that psychotic symptoms may be meaningfully related to this woman's depression and potentially affected or were influenced by her perfectionism. This case, along with the existing literature linking psychotic symptoms with suicide, identifies an area that has not been yet researched and yet may be instructive in understanding suicide and ways to intervene with those experiencing psychotic symptoms: specifically, the area of perfectionism, psychotic symptoms, and suicide. For those who are already at risk for suicide because of their psychotic experiences, especially those experiencing psychotic symptoms for the first time, perfectionistic tendencies may magnify their risk, making them particularly vulnerable to completed suicide.

The young woman described herein experienced a high degree of hopelessness, chronic depression, and almost no self-esteem, including negative social comparisons with others and high nondisplay of imperfection. She is someone who viewed herself as wholly and permanently imperfect and flawed, and who did not want anyone to see it. Her suicide suggests it may be important to assess perfectionism and include it as a clinical focus in the therapeutic context. Future research in this area may elucidate the role of perfectionism in suicide, singularly and in the context of a comprehensive clinical risk assessment, demonstrating whether perfectionism confers information about suicide risk beyond known clinical risk factors. Research into the relationship among perfectionism, psychotic symptoms, and suicide may shed light on whether there is any clinical utility in addressing perfectionistic tendencies in those experiencing psychotic symptoms. With deeper understanding of how perfectionism may interact with other risk factors for suicide, additional research may explore whether perfectionistic tendencies, including cognitive rigidity, may be effectively addressed and possibly reduce the impact of perfectionism on other known risk factors for suicide.

## References

[1] Blatt SJ (1995). The destructiveness of perfectionism: implications for the treatment of depression. *The American Psychologist*.

[2] Flett GL, Hewitt PL, Heisel MJ The destructiveness of perfectionism revisited: Implications for the assessment of suicide risk and the prevention of suicide.

[3] Hewitt PL, Flett GL (1991). Perfectionism in the self and social contexts: conceptualization, assessment, and association with psychopathology. *Journal of Personality and Social Psychology*.

[4] Flett GL, Hewitt PL, Garshowitz M, Martin TR (1997). Personality, negative social interactions, and depressive symptoms. *Canadian Journal of Behavioural Science*.

[5] Hassan S (2011). *Perfectionism, distress tolerance, emotional self-efficacy, and psychological distress: an analysis of emotional perfectionism [M.S. thesis]*.

[6] Stoeber J, Harris RA, Moon PS (2007). Perfectionism and the experience of pride, shame, and guilt: comparing healthy perfectionists, unhealthy perfectionists, and non-perfectionists. *Personality and Individual Differences*.

[7] Hewitt PL, Flett GL, Sherry SB, Caelian C, Ellis TE (2006). Trait perfectionism dimensions and suicidal behavior. *Cognition and Suicide: Theory, Research, and Therapy*.

[8] Roxborough HM, Hewitt PL, Kaldas J (2012). Perfectionistic self-presentation, socially prescribed perfectionism, and suicide in youth: a test of the perfectionism social disconnection model. *Suicide and Life-Threatening Behavior*.

[9] Hewitt PL, Flett GL, Sherry SB (2003). The interpersonal expression of perfection: perfectionistic self-presentation and psychological distress. *Journal of Personality and Social Psychology*.

[10] Harkavy-Friedman JM (2006). Can early detection of psychosis prevent suicidal behavior?. *The American Journal of Psychiatry*.

[11] Robinson J, Harris MG, Harrigan SM (2010). Suicide attempt in first-episode psychosis: a 7.4 year follow-up study. *Schizophrenia Research*.

[12] Demerath NJ (1943). Adolescent status demands and the student experiences of twenty schizophrenics. *American Sociological Review*.

[13] Rector NA (2004). Dysfunctional attitudes and symptom expression in schizophrenia: differential associations with paranoid delusions and negative symptoms. *Journal of Cognitive Psychotherapy*.

[14] Hassan S, Ganguli R, Flett GL, Hewitt PL (2013). An exploratory examination of perfectionistic self-presentation in psychosis. *Schizophrenia Bulletin*.

[15] Hassan S, Ganguli R, Flett GL, Hewitt PL An exploratory examination of perfectionistic self-presentation in psychosis.

[16] Fialko L, Freeman D, Bebbington PE (2006). Understanding suicidal ideation in psychosis: findings from the Psychological Prevention of Relapse in Psychosis (PRP) trial. *Acta Psychiatrica Scandinavica*.

[17] American Psychiatric Association (1987). *Diagnostic and Statistical Manual of Mental Disorders*.

[18] Overall J, Gorham D (1988). The brief psychiatric rating scale: recent developments in ascertainment and scaling. *Psychopharmacology Bulletin*.

[19] Andreasen NC (1982). Negative symptoms in schizophrenia: definition and reliability. *Archives of General Psychiatry*.

[20] Addington D, Addington J, Schissel B (1990). A depression rating scale for schizophrenics. *Schizophrenia Research*.

[21] Rosenberg M (1965). *Society and the Adolescent Self-Image*.

[22] Guy W, Guy W (1976). Clinical global impressions. *ECDEU Assessment Manual for Psychopharmacology*.

[23] Hewitt PL, Flett GL, Turnbull-Donovan W, Mikail SF (1991). The multidimensional perfectionism scale: reliability, validity, and psychometric properties in psychiatric samples. *Psychological Assessment*.

[24] Hewitt PL, Flett GL, Turnbull-Donovan W (1992). Perfectionism and suicide potential. *British Journal of Clinical Psychology*.

[25] Andrews B, Qian M, Valentine JD (2002). Predicting depressive symptoms with a new measure of shame: the experience of shame scale. *British Journal of Clinical Psychology*.

[26] Dearing RL, Tangney JP (2011). *Shame in the Therapy Hour*.

[27] Lester D (1997). The role of shame in suicide. *Suicide and Life-Threatening Behavior*.

[28] Joiner TE, Pettit JW, Walker RL (2002). Perceived burdensomeness and suicidality: two studies on the suicide notes of those attempting and those completing suicide. *Journal of Social and Clinical Psychology*.

[29] Rasmussen KA, Slish ML, Wingate LRR, Davidson CL, Grant DMM (2012). Can perceived burdensomeness explain the relationship between suicide and perfectionism?. *Suicide and Life-Threatening Behavior*.

[30] Kernberg O (1975). *Borderline Conditions and Pathological Narcissism*.

[31] Kernberg O (1976). *Object Relations Theory and Clinical Psychoanalysis*.

[32] Apter A, Bleich A, King RA (1993). Death without warning? A clinical postmortem study of suicide in 43 Israeli adolescent males. *Archives of General Psychiatry*.

[33] Arie M, Haruvi-Catalan L, Apter A (2005). Personality and suicidal behavior in adolescence. *Clinical Neuropsychiatry*.

[34] Bieling PJ, Summerfeldt LJ, Israeli AL, Antony MM (2004). Perfectionism as an explanatory construct in comorbidity of axis I disorders. *Journal of Psychopathology and Behavioral Assessment*.

[35] Beck AT, Brown G, Steer RA (1989). Prediction of eventual suicide in psychiatric inpatients by clinical ratings of hopelessness. *Journal of Consulting and Clinical Psychology*.

[36] Beck AT, Steer RA, Kovacs M, Garrison B (1985). Hopelessness and eventual suicide: a 10-year prospective study of patients hospitalized with suicidal ideation. *The American Journal of Psychiatry*.

[37] Rizwan M, Ahmad R (2010). Self-esteem as a predictor of suicide risk among psychiatric patients. *Journal of Alternative Perspectives in the Social Sciences*.

[38] Moorhead S, Samarasekera N, Turkington D (2005). Schemas, psychotic themes and depression: a preliminary investigation. *Behavioural and Cognitive Psychotherapy*.

